# Pathological characteristics and immune microenvironment of SMARCA4-deficient undifferentiated uterine sarcoma

**DOI:** 10.1186/s13000-023-01347-3

**Published:** 2023-05-16

**Authors:** Jie Gao, Ruirui Fan, Dahong Chen, Jinlin Hou, Hanlin Chen, Mingzhi Lu

**Affiliations:** 1 Department of Pathology, Xiamen Humanity Hospital Fujian Medical University, No.3777 Xianyue Street, Xiamen, Fujian 361000 China; 2 Department of Pathology, Xiang’an Hospital of Xiamen University, School of Medicine, Xiamen University, Xiamen, China; 3 Department of Gynecology, Xiamen Humanity Hospital Fujian Medical University, Xiamen, China; 4 Nanjing Geneseeq Technology Inc, Nanjing, China

**Keywords:** Undifferentiated uterine sarcoma, *SMARCA4-*deficient undifferentiated uterine sarcoma, *SMARCA4*, Immune microenvironment, NGS analysis

## Abstract

*SMARCA4-*deficient undifferentiated uterine sarcoma (SDUS) is a highly invasive single-gene malignant tumor caused by mutations in the *SMARCA4* gene. SDUS has a poor prognosis, with no established treatment strategy at present. Further, there is a lack of relevant research on the role of the immune microenvironment in SDUS worldwide. Here, we report a case of SDUS that was diagnosed and analysed using morphological, immunohistochemical, and molecular detection techniques, along with the analysis of the immune microenvironment. By immunohistochemistry, the tumor cells showed retained INI-1 expression, focal CD10 expression, and loss of BRG1, CK-pan, synaptophysin, desmin, and ER expression. Further, some of the immune cells expressing CD3 and CD8 had infiltrated into the SDUS, but no PD-L1 expression was detected. The multiple immunofluorescent staining results showed that a proportion of the immune cells and SDUS cells expressed CD8/CD68/PD-1/PD-L1. Therefore, our report will help in the diagnostic awareness of SDUS.

## Introduction

Uterine sarcomas account for 3–5% of all uterine tumors with an incidence rate of 1.55–1.95 per 100,000 women per year [1]. The inactivation mutation of the *SMARCA4* gene in this sarcoma was first discovered and proposed by Kolin et al. in 2018^2^. *SMARCA4-*deficient undifferentiated uterine sarcoma (SDUS) is a highly aggressive undifferentiated uterine sarcoma caused by a *SMARCA4* deletion. Here, *SMARCA4* acts as the main driving gene and SDUS has a low tumor mutation burden [2]. *SMARCA4* is involved in many cellular processes, such as the transcription-related regulation, DNA damage repair, and is usually found in the promoters of transcriptional genes. As such, the changes in the transcriptional and epigenetic regulations caused by *SMARCA4* loss may play a central role in driving tumorigenesis [3].

To date less than 30 cases of SDUS have been reported worldwide. In these cases, the average age of the patients was 36 years, which is much younger than that of patients with undifferentiated endometrial cancer (average 61 years) [1], but slightly older than that of patients with small cell carcinoma of the ovary, hypercalcaemic type (SCCOHT) (average 29 years) [4]. Since SDUS is highly invasive and has no established treatment strategy at present, despite active surgical interventions, the prognosis is extremely poor with a median survival time of only 9 months [5]. SUDS did not express CK, CD56, CgA, Syn, EMA and other cancer markers, but expressed Vimintin. Presently, the diagnosis of SDUS is mainly based on the results of morphological analysis, and the observation of BRG1 loss and *SMARCA4* gene deletion. Morphologically, SDUS is composed of atypical, epithelioid cells with prominent rhabdoid morphology, and, therefore, sometimes difficult to distinguish from the SCCOHT [6].

## Case report

**Fig. 1:**
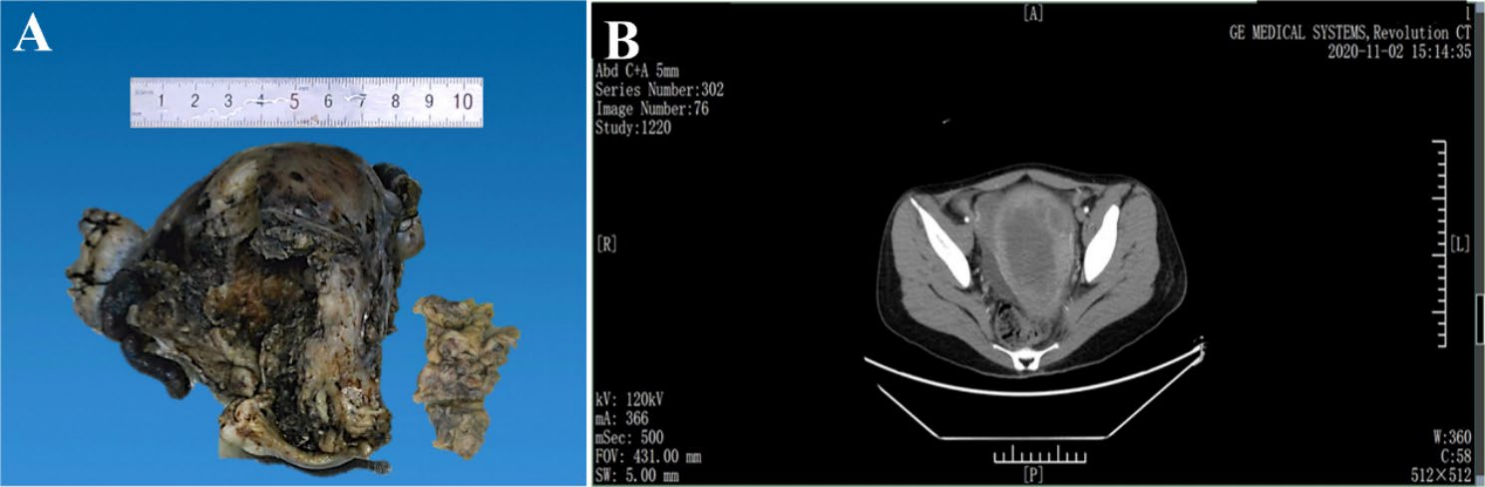
(A) Gross specimens of whole uterus and tumor. (B) Computed tomography shows a space occupying lesion in the uterus

A 27-year-old Chinese woman presented with menorrhagia for three years and vaginal bleeding for 24 days. Enhanced arterial phase computed tomography results showed that a significant increase in the uterine volume, and space-occupying lesions with a size of approximately 9.5 cm × 8.5 cm × 8 cm were observed. A mixed density shadow was observed in the uterus, with an unclear boundary **(**Fig. 1-A**)**. The size of the uterus was 12 cm × 10 cm ×9 cm, the depth of the uterine cavity was 8 cm, and the thickness of the endometrium was 0.2–0.3 cm. There was a large mass in the uterine cavity with a size of 9 cm × 8 cm × 8 cm; the section was grey white and grey red. No SDUS tumor involving double appendages was found during enhanced CT and surgery **(**Fig. 1-B**).**

During surgery, diffuse uterine enlargement was observed, resembling the size at approximately 16 weeks of gestation; the surface of the uterus had a hard texture with many hard nodules, each with a diameter of approximately 1 cm. When a cut was made into the uterine cavity, the lesion had no boundary with the normal myometrium and were easily broken. The adhesion between the left bladder and the lower uterine segment was dense, and the tumor had infiltrated into the serosal surface of the lower-left anterior wall of the uterus. No lesions were observed in the bilateral appendages or other organs.

**Fig. 2:**
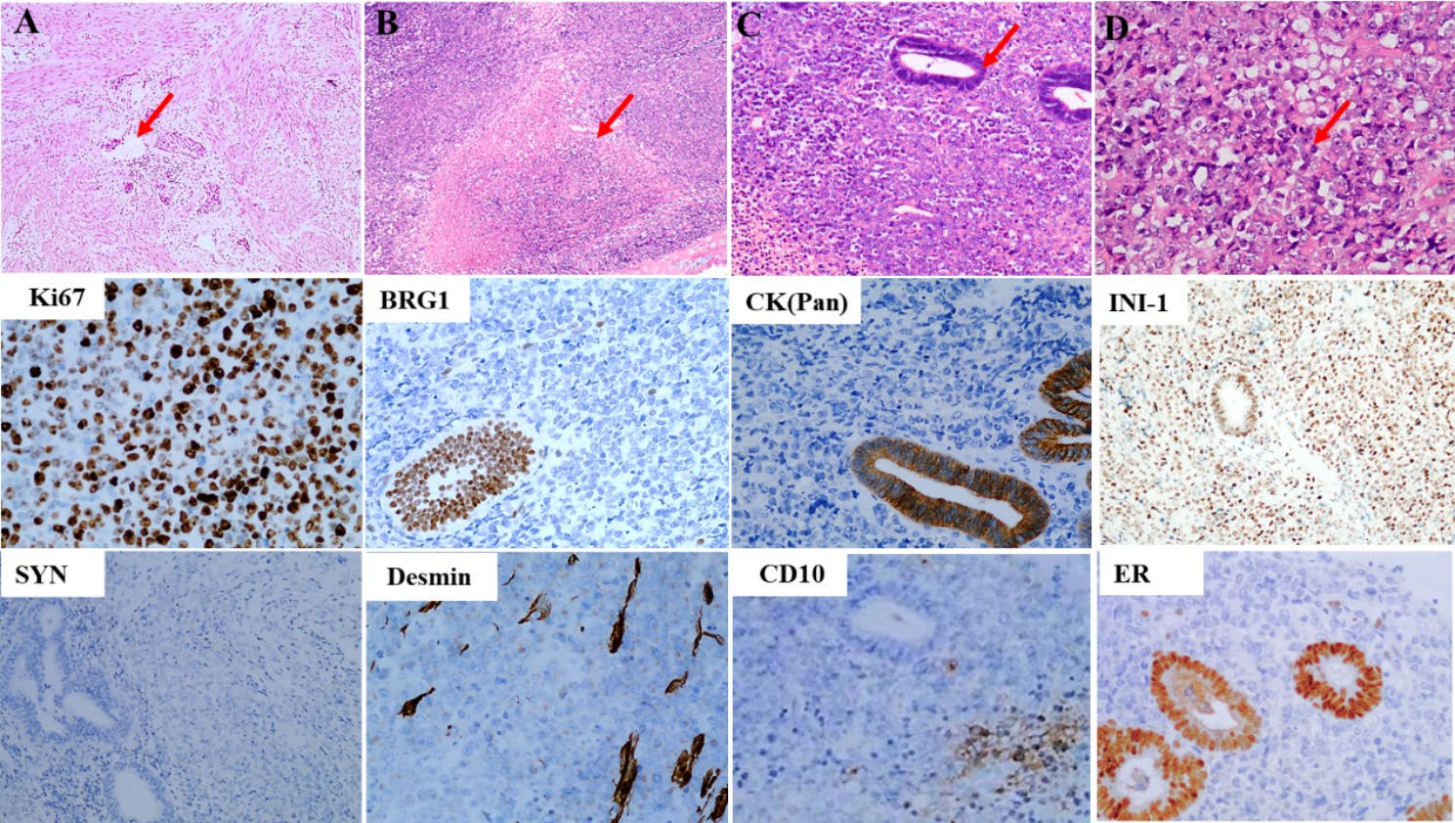
Hematoxylin-eosin and immunohistochemical staining of SDUS lesions. (A) Tumor cells invading blood vessels (40×, red arrow); (B) Tumor necrosis area (40×); (C) Tumor involved normal endometrial glands with tumor necrosis (100×); (D) Tumor cells were epithelioid medium-to-large cells with local rhabdoid morphology (200×). Immunohistochemical staining for the Ki-67 proliferation marker immunohistochemistry showing a high percentage (60%) of positively stained cells, along with the observation of INI-1 expression, local of expression of CD10, and the lack of expression of BRG1, CK, SYN, desmin, and ER (200×)

Hematoxylin and eosin staining was performed. Microscopically, the tumor cells were diffusely distributed, displaying flake-like or acinar morphology. SDUS tumor cells in some areas had lost adhesion, and some SDUS tumor tissues grew around and invaded the blood vessels **(**Fig. 2-A**)**. Extensive necrosis was observed **(**Fig. 2-B**).** Residual benign endometrial glands were observed **(**Fig. 2-C**).** The tumor cells were epithelioid to rhabroid with eccentric round or oval nuclei and eosinophilic cytoplasm **(**Fig. 2-D**)**. By immunohistochemistry, the tumor cells showed a high (60%) Ki-67 proliferation index, retained INI-1 expression, wild-type p53 expression, focal CD10 expression, and loss of the expression of BRG1, CK-pan, synaptophysin, desmin, and ER **(**Fig. 2**)**.

**Fig. 3:**
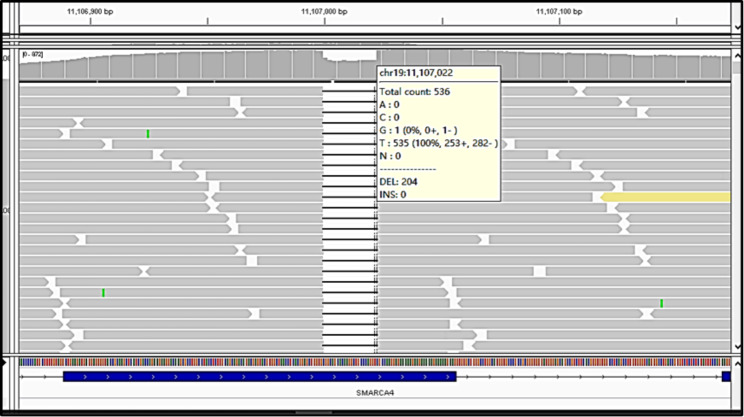
Next-generation sequencing analysis showing a deletion mutation in *SMARCA4*

Next-generation sequencing (NGS) was carried out (Nanjing, China). Formalin-fixed paraffin-embedded sections of the uterine tumor were subjected to comprehensive NGS analysis with 425 predefined related genes. Data were sequentially analysed by with the use of appropriate bioinformatics tools. Germline mutations were filtered through comparison of the data with those of the patient’s whole blood controls. The genetic analysis of our patient detected no germline mutation in her family. A SMARCA4 splicing mutation, c.355 + 190_616del, was detected at a MAF of 86.4% in the tumor sample, accompanied by a SMARCA4 frameshift mutation p.H571Gfs (45.8%). The tumor mutation burden (TMB) was 1.1 mutations/Mb **(**Fig. 3**).** The NGS analysis included the investigation of important markers, such as MMR, MSI, *PTEN, PIK3CA, TP53, beta-catenin*, etc. Our NGS test report showed *SMARCB1, CTNNBI, MMR, MSI, PTEN, PIK3CA, TP53* were negative.

Finally, based on the genetic analysis histomorphology and immunostaining results, a clinical diagnosis of SDUS was confirmed following consultation with an expert.

**Fig. 4:**
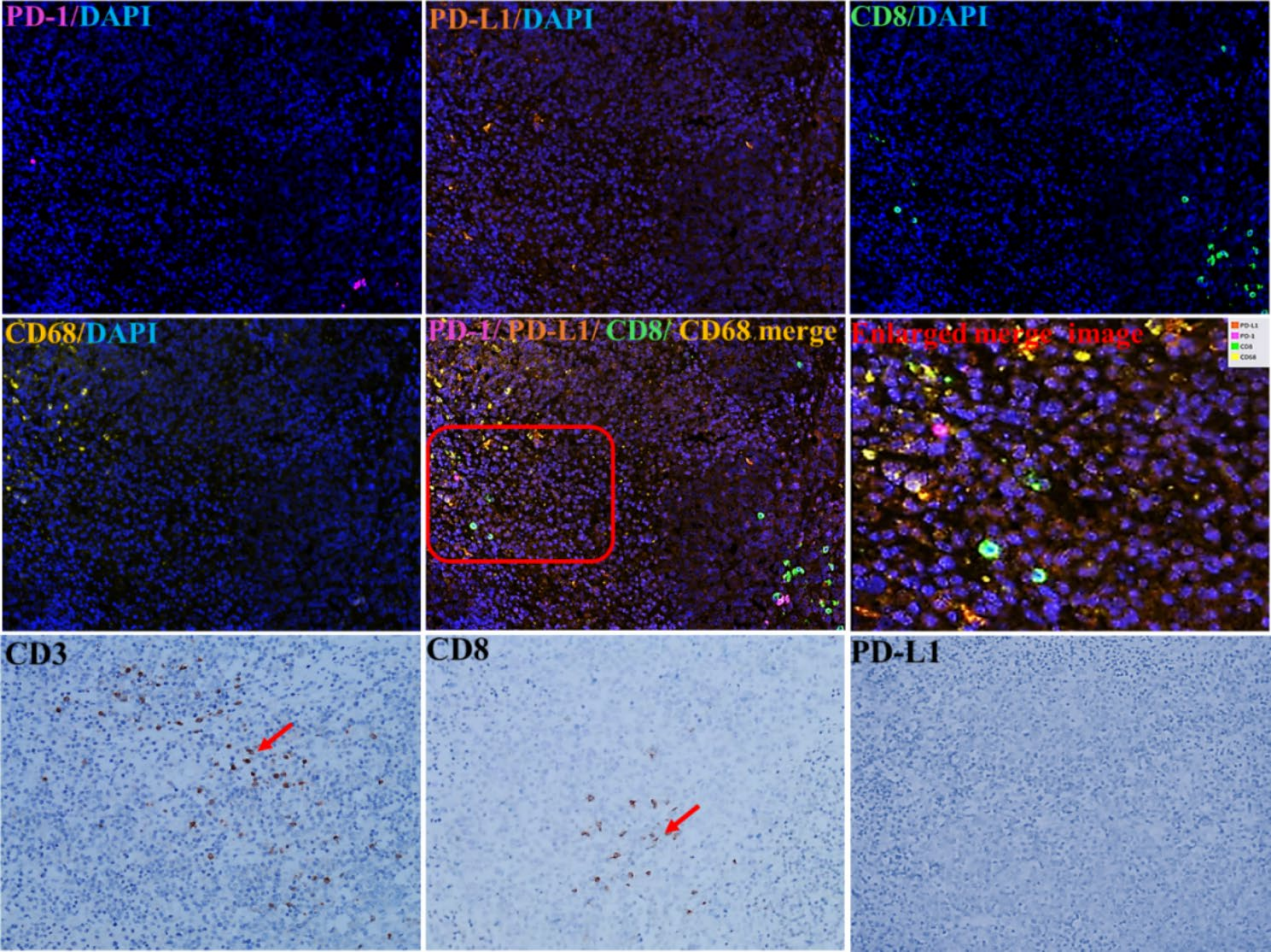
Results of multiple immunofluorescence analysis showed that immune cells and SDUS cells expressed CD8/CD68/PD-1/PD-L1 (100×). Immunohistochemical results showed that some of the immune cells expressing CD3(100×) and CD8(100×) had infiltrated into SDUS (Red arrow), but no PD-L1(40×) expression was detected

The multiple immunofluorescent staining of the SDUS lesions was performed at Genecast Biotechnology Co. Ltd. (Beijing, China). Briefly, 4-µm-thick sections were sliced from SDUS tissues for analysis. The tissue sections were incubated with primary antibodies against PD-L1 (1:25), CD68 (1:500), and PD-1 (1:50) for 1 h at 25℃, and with primary antibodies against CD8 (1:100) and CD3 (1:100) overnight at 4℃. The results showed that there were some infiltrating immune cells expressing CD8(+,0.92%) and CD68(+,0.2%) in SDUS tissues. A few immune cells and SDUS cells expressing PD-1(+,0.06%) and PD-L1(+,0.06%) were also detected by immunofluorescence **(**Fig. 4**).** Immunohistochemical results showed that some of the immune cells expressing CD3 and CD8 had infiltrated into SDUS tissues, but no PD-L1 expression was detected **(**Fig. 4**).** Approximately 27 T-cells per high power field (HPF) had infiltrated into the SDUS tissues. The antibody information is presented in Table 1.

**Table 1:** Antibody clones, and suppliers used for immune stains

Antibody	Clone	Supplier
BRG1	E8V5B	ZSGB-BIO, Co.Ltd,China
CD10	MX002	Fuzhou Maixin biotech.Co.Ltd,China
ER	SP1	Fuzhou Maixin biotech.Co.Ltd,China
Myogenin	F5D	Fuzhou Maixin biotech.Co.Ltd,China
Desmin	MX046	Fuzhou Maixin biotech.Co.Ltd,China
CKpan	AE1/AE3	Fuzhou Maixin biotech.Co.Ltd,China
Ki67	MX006	Fuzhou Maixin biotech.Co.Ltd,China
Syn	MX038	Fuzhou Maixin biotech.Co.Ltd,China
SMA	1A4	Fuzhou Maixin biotech.Co.Ltd,China
CD34	QBEnd/10	Fuzhou Maixin biotech.Co.Ltd,China
CD3	SP7	Fuzhou Maixin biotech.Co.Ltd,China
CD8	SP16	Fuzhou Maixin biotech.Co.Ltd,China
CD99	O13	Fuzhou Maixin biotech.Co.Ltd,China
INI1	MRQ-27	Fuzhou Maixin biotech.Co.Ltd,China
PD-L1	22C3	Dako, Carpinteria, CA, USA
PD1	ZM-0381	ZSGB-BIO, Co.Ltd,China
CD68	ZM-0060	ZSGB-BIO, Co.Ltd,China
Calponin	MX023	Fuzhou Maixin biotech.Co.Ltd,China

**Fig. 5:**
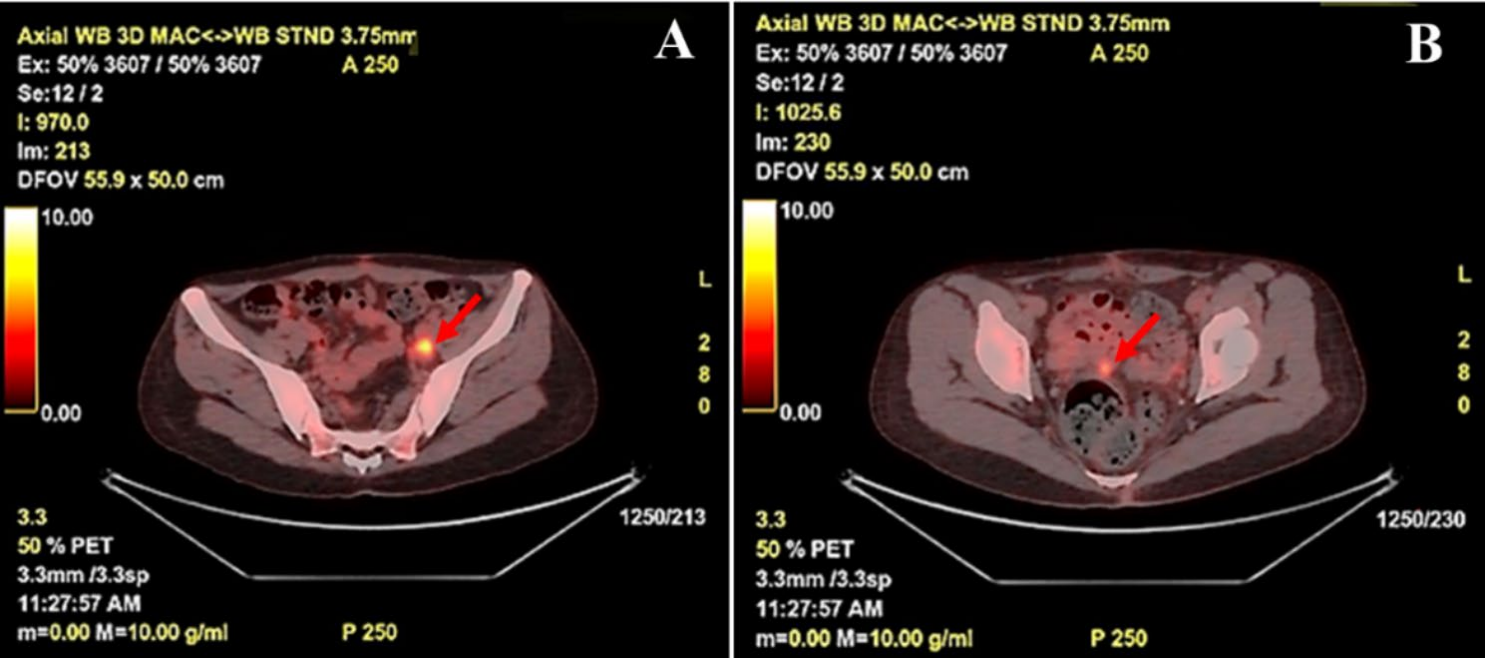
PET-CT showed hypermetabolic foci in the left iliac fossa (A) and pelvis(B)

In our patient, the tumor stage was pT3bNxMx. The patient underwent hysterectomy and bilateral salpingo-oophorectomy, and then received 4 cycles of gemcitabine and docetaxel chemotherapy. Positron emission tomography–CT (PET-CT) examination showed hypermetabolic signals in the left iliac fossa and pelvic cavity of the patient, suggesting that the tumor showed a trend toward further deterioration **(**Fig. 5**)**. Thus, the treatment plan was changed to epirubicin and ifosfamide chemotherapy. Following 3 cycles of treatment, the patient eventually gave up on chemotherapy due to severe side effects. The patient is still alive and showed an enlargement of the left iliac fossa lesion during the 6-month follow-up.

## Discussion

There are currently less than 30 SDUS cases published worldwide [1]. Although active surgical treatment is performed, the prognosis is poor. Due to the rarity and specificity of SDUS, there is no established treatment at present. Therefore, accurate diagnosis is of great significance for the prognosis of patients. Pathologists especially need to differentiate SDUS from the following types of similar tumors.

SCCOHT is usually a huge, unilateral mass (average 15 cm), while SDUS may involve the bilateral adnexa [7]. There are also differences in clinical manifestations due to different tumor origins: SDUS often presents with cervical mass or vaginal bleeding, while SCCOHT most commonly manifests as abdominal pain or as an abdominal mass, with less cervical mass or vaginal bleeding [7]. By immunohistochemistry, SCOOHT showed the expression of CD56, CgA, Syn, CK (Pan), EMA and other cancer markers, but SDUS is not expressed. Histologically, epithelioid uterine leiomyosarcomas show notable nuclear pleomorphism, but SDUS cells have more consistent atypia without apparent pleomorphism. Immunohistochemically, the detection of BRG1 deletion can effectively exclude leiomyosarcoma [8]. At the molecular level, leiomyosarcoma often has no *SMARCA4* deletion mutation. Endometrial stromal sarcomas cells usually do not have rhabdoid morphology. Immunohistochemistry shows that CD10 is strongly expressed. CyclinD1, which is strongly expressed, is found in high-grade stromal sarcoma. These markers are not found in the SDUS. At the molecular level, endometrial stromal sarcomas often have no *SMARCA4* deletion mutation. Further, any mutations associated with endometrial mesenchymal sarcoma were not found in our NGS test. As for distinguishing SDUS from undifferentiated endometrial carcinoma, SDUS often has a lobulated structure [9], fewer *TP53* mutations, and is characterized by inactivation mutations in *SMARCA4*^10^. In contrast to undifferentiated endometrial carcinomas, SDUS frequently has some residual benign endometrial glands, and it is microsatellite-stable with low expression of Claudin-4/CK/EMA. The average age of patients with SDUS is 36 years, which is younger than that of patients with undifferentiated endometrial cancer [5]. Although the onset ages of proximal epithelioid sarcomas and SDUS are similar, there are some differences. First, SDUS occurs in the uterus, whereas proximal epithelioid sarcomas are usually found in the groin or vulva. Second, immunohistochemistry shows that CD34 is expressed in more than 50% of epithelioid sarcomas, but a lack of CD34 expression is observed in SDUS [10]. Finally, while *SMARCB1* deletion is a typical feature of epithelioid sarcoma, the *SMARCA4* deletion mutation is only found in SDUS [10].

Presently, the targeted therapy of SCCOHT entails several approaches [11]–[12], and studies have shown immunotherapy to be the best choice for treatment [3]. Jelinic P found unexpectedly that for a low mutation burden cancer(SCCOHT), the majority of the tumors (eight of 11 cases) demonstrated PD-L1 expression with strong associated T-cell infiltration (R2 ¼ 0.60–0.95) [13]. These data suggest that SCCOHTs are immunogenic tumors. They also believed that the transcriptional program regulated by SMARCA4 may influence tumor immunogenicity, leading to TIL infiltration and PD-L1 upregulation. As SCCOHT and SDUS show several similarities in terms of their morphological characterization and the gene mutations involved and as both are low mutation load tumors, future SDUS treatments may take direction from current SCCOHT treatments. At present, there is no established treatment and there is no relevant research on the role of the immune microenvironment in SDUS. Accordingly, we examined the expression of CD3, CD8, PD-1, and PD-L1 in SDUS tumor cells and further explored the tumor immune microenvironments. Our immunoassay results showed that approximately 27 CD8+/CD3 + T-cells per high power field (HPF) had infiltrated into SDUS tissues. The results showed this case of is different from the previously reported SCCOHT, and its immunogenicity is weak. Therefore, the patient did not receive immunotherapy in the end.

Our study has some limitations. First of all, this is the first time to study the immune microenvironment of SDUS, but since there is only one case, the difference in immunogenicity between SDUS and SCCOHT cannot be determined. In the future, we hope to find more SDUS cases for the study of the immune microenvironment. Second, no germline mutation was detected in her family. Identification of cases of malignant tumors with a *SMARCA4* deletion in the uterus is important; investigation of the role of the immune microenvironment from various perspectives in SDUS is needed.

## Conclusion

SDUS poses a diagnostic challenge as little data concerning its histology, presentation during a long-term follow-up and outcome are available. Further clinicopathological and molecular cytogenetic studies of more cases are needed to further characterize SDUS.
